# Workplace-Related Traumatic Injuries: Insights from a Rapidly Developing Middle Eastern Country

**DOI:** 10.1155/2014/430832

**Published:** 2014-03-05

**Authors:** Hassan Al-Thani, Ayman El-Menyar, Husham Abdelrahman, Ahmad Zarour, Rafael Consunji, Ruben Peralta, Mohammad Asim, Hany El-Hennawy, Ashok Parchani, Rifat Latifi

**Affiliations:** ^1^Trauma Surgery Section, Hamad General Hospital, P.O. Box 3050, Doha, Qatar; ^2^Clinical Research, Trauma Surgery Section, Hamad General Hospital, P.O. Box 3050, Doha, Qatar; ^3^Clinical Medicine, Weill Cornell Medical College, P.O. Box 24144, Doha, Qatar; ^4^Department of Surgery, Weill Cornell Medical College, P.O. Box 24144, Doha, Qatar; ^5^Department of Surgery, University of Arizona, P.O. Box 245005, Tucson, AZ, USA

## Abstract

Traumatic workplace-related injuries (WRIs) carry a substantial negative impact on the public health worldwide. We aimed to study the incidence and outcomes of WRIs in Qatar. We conducted occupational injury surveillance for all WRI patients between 2010 and 2012. A total of 5152 patients were admitted to the level 1 trauma unit in Qatar, of which 1496 (29%) sustained WRI with a mean age of 34.3 ± 10.3. Fall from height (FFH) (51%) followed by being struck by heavy objects (FHO) (18%) and motor vehicle crashes (MVC) (17%) was the commonest mechanism of injury (MOI). WRI patients were mainly laborers involved in industrial work (43%), transportation (18%), installation/repair (12%), carpentry (9%), and housekeeping (3%). Use of protective device was not observed in 64% of cases. The mean ISS was 11.7 ± 8.9, median ICU stay was 3 days (1–64), and total hospital stay was 6 days (1–192). The overall case fatality was 3.7%. Although the incidence of WRI in Qatar is quite substantial, its mortality rate is relatively low in comparison to other countries of similar socioeconomic status. Prolonged hospital stay and treatment exert a significant socioeconomic burden on the nation's and families' resources. Focused and efficient injury prevention strategies are mandatory to prevent future WRI.

## 1. Introduction

Workplace-related traumatic injuries (WRIs) account for a significant proportion of injuries that affect mainly the young population [1]. These injuries are a major source of disability and death leading to serious socioeconomic losses to the family of victims [2]. According to the International Labor Organization (ILO), the global mortality (2008) from fatal occupational accidents was 321,000 [3]. In addition, around 317 million workers sustain nonfatal WRI annually, leading to an average loss of productivity of four days or more per year [3].

Most epidemiological information regarding WRI is derived from developed and industrialized countries. An Australian report on fatal WRI observed 374 deaths, a mortality rate of 1.93 per 100 000 workers, in 2010-2011. Of these, 59% were directly job-related, 29% were transport-related, and 12% were bystander fatalities [4]. Data from Singapore showed that the majority of WRI patients were admitted due to fall from height (FFH) (66%) and falling of heavy objects (FHO) (21.9%) at work with a case fatality of 4.7% [5].

International migrants and migrant workers have contributed significantly to the economic growth and rapid development needs of several countries [3]. A review conducted on occupational injury and diseases among immigrant workers found that immigrant workers had a disproportionately higher burden of occupation-related morbidity and mortality in comparison to native workers [6]. Other studies reported an increased risk of occupational disability among construction workers compared to those workers involved in less physical labor [7, 8]. Further, Gubéran and Usel proposed a higher risk of occupational disability in unskilled workers in comparison to the skilled workers [7]. Among construction site injuries, FFH is the most frequent cause of injury [9].

In the Middle East, the majority of fall-related injuries are associated with the construction and petrochemical industries. The rate of fall-related injuries is markedly increased in rapidly developing Middle Eastern countries with increasing demands for construction work. There are only few reports from the Middle East that describe the pattern of injury and safety measures in those who sustained traumatic injuries purely from FHO at the construction sites [10]. In UAE, the incidence of occupational injury was reported as 136/100,000 workers per year [11]. The main causes of such injuries were FFH (51%) and FHO (15%) [10–12].

Traumatic injuries cause a major burden on the healthcare system of Qatar. A booming economy and rapid industrialization in Qatar attract thousands of skilled and unskilled workers from South Asia and other countries. The state of Qatar is a rapidly developing, high-income country with a population that is 75% male and is 85% expatriates [13]. Its rapid economic growth has been fueled by its vast petrochemical reserves and accompanied by massive infrastructure projects, most notably for the World Cup in 2022, which have necessitated the rapid influx of thousands of migrant workers leading to doubling of its population in the last 7 years. However, there are very few published reports on WRI in Qatar. The incidence and mortality from construction that fall in Qatar between 2007 and 2008 were reported as 86.7 and 8.44 per 100,000 construction workers [10]. Most recently, this worker movement has been accompanied by an Amnesty International Report that discusses worker rights abuses but does not specify WRI as a main issue [14]. Herein, we aim to describe the incidence and outcomes of WRI in Qatar, a rapidly developing country in the Middle East between 2010 and 2012.

## 2. Methods

A descriptive analysis of trauma registry data for occupational injury surveillance was conducted; this included all WRI patients admitted at level I Hamad Trauma Center (the national trauma referral center and only provider of tertiary trauma care in Qatar), from January 1, 2010, to December 31, 2012. Labor statistics from the Qatar Statistics Authority were used to compute for the population based incidence and mortality rates from WRI in Qatar [13]. Data analyses included demographics, clinical presentations, mechanism of injury (MOI), injury severity score (ISS), type of occupation, use of protective measures, hospital length of stay, need for rehabilitation facility, morbidity, and mortality. Data were also analyzed according to the different mechanisms of injury.

### 2.1. Definitions

*WRIs* were defined as injuries taking place during the specified working hours, while the employee is engaged in work-related activities [15]. *Pedestrian injuries* were included if related to work. *Injury burden *is the proportion of injuries caused by a specific injury mechanism among the total number of workplace-related injuries. *Mortality burden* is the proportion of deaths caused by a specific injury mechanism among the total number of deaths due to WRI. *Case fatality rate* is the number of deaths caused by a specific injury mechanism divided by the total number of workplace-related injuries caused by that specific mechanism of injury.

Based on a mandate from the Supreme Council of Health in Qatar, all emergency medical services (prehospital and in-hospital services) are provided free of charge for all Qatari nationals and residents, including expatriate workers. Moreover, Hamad Medical Corporation is responsible for providing rehabilitation services, free of charge, for all patients until they are discharged home. The majority of migrant workers return to their home countries after serious injuries or disabilities, if they have no family support in Qatar.

To compute for the ISS, each of six anatomical regions was scored with the highest Abbreviated Injury Score (AIS) using the square of the highest value of the three most severely injured body regions. The total score ranged from 1 to 75 [16].

Ethical approval was obtained from the Medical Research Center (IRB number 13186/13) at Hamad Medical Corporation, Doha, Qatar.

Data were presented as proportions, medians, or mean ± standard deviation (SD), as appropriate. Differences in categorical variables between respective comparison groups were analyzed using Chi-Square test. The continuous variables were analyzed using one-way ANOVA. Two tailed *P* values < 0.05 were considered to be significant. Data analysis was carried out using the Statistical Package for the Social Sciences version 18 (SPSS Inc., Chicago, IL, USA).

## 3. Results

During the 3-year surveillance, a total of 5152 patients were admitted at the trauma unit, of them 1496 (29%) sustained WRI with a mean age of 34 ± 10 years and 97% of them were males. Age breakdown revealed that 72% of patients were young (20 to 40 years) (Table 1). The majority of the patients were from South Asian countries particularly from Nepal (29%) and India (21%). FFH (51%), FHO (18%), and motor vehicle crashes (MVCs) (17%) were the most common MOI. WRI patients were mainly laborers involved in industrial work (43%), transportation (18%), installation/repair (12%), carpentry (9%), and housekeeping (3%). Sixty-four percent of WRI patients were not using personal protective equipment (PPE) when they were injured. The lower extremities (28%) followed by chest (26%), upper extremities (23%), head (20.5%), and abdomen (18%) were the most frequently injured body regions. There were 308, 161, and 92 spinal injuries in terms of lumber, thoracic, and cervical vertebrae, respectively. Open reduction and internal fixations (ORIF) (27%) were the most commonly performed surgical procedure followed by exploratory laparotomy (6%), closed reduction (5%), and craniotomy (3%). The study population had median ventilator days of 2 (1–21), ICU stay of 3 (1–64), and total hospital stay of 6 (1–192) days. Forty-nine percent of the WRI patients stayed more than 7 days in the hospital and 13.6% were discharged to long-term rehabilitation facilities. The mean injury severity score (ISS) was 11.7 ± 8.9 and Glasgow coma score was 14.03 ± 2.8 (Table 2).

**Figure 1:**
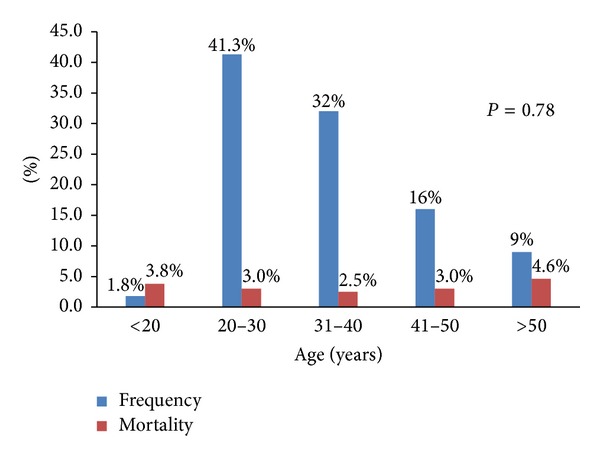
Percentage distribution of WRI and case fatality in different age groups.

The overall mortality was 3.7%. Prehospital deaths (BID) constituted 40.5% of all mortalities which included mortality either at the workplace or during transportation to the hospital. The main MOI of BID was FFH (41%) followed by MVCs (23%), FHO, machinery, burn, and pedestrian injuries (9% for each). More young workers died but the case fatality was nonsignificantly higher for older (>50 years) compared to young workers (Figure 1).

Table 3 shows that FFH represents the highest injury (51%) and mortality (38%) burden among the MOI, whereas the case fatality was high among burn (17 per 100 cases) and pedestrian (12 per 100 cases) injuries.

**Figure 2:**
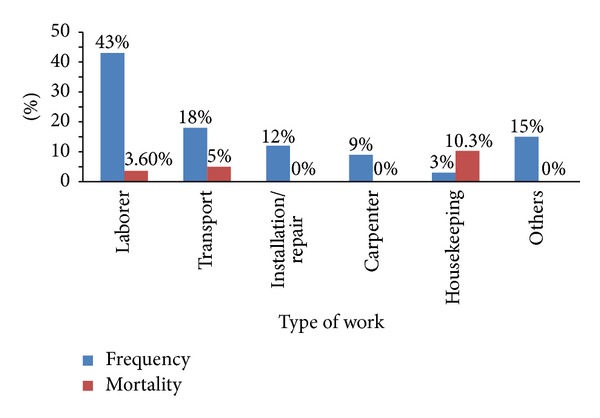
Percentage distribution of WRI and case fatality according to type of work.

Figure 2 demonstrates the proportion of injuries and case fatality according to type of work. Most WRI affected construction laborers and workers involved in transport and installers/repairmen job but the highest mortality rate was observed in workers involved in housekeeping jobs.

Table 4 represents the demographic characteristics, presentation, and outcome of WRI patients by MOI. Laborers were disproportionately highly involved in injuries related to machines, FHO and pedestrian injuries, respectively. Not surprisingly, almost all WRI due to MVC involved transport occupations. Installation/repair workers represented around 12–16% of patients in all MOI except burns (31%) and MVC (0%). Housekeeping workers were involved mainly in injuries due to burns followed by FFH. In comparison to other MOI, FFH was more likely associated with lack of PPE use (*P* = 0.001) and head (*P* = 0.002), thoracic (*P* = 0.001), and lumbar spine (*P* = 0.001) injuries. Pedestrian injuries were characterized by higher mean ISS and more frequently associated with chest, abdomen, pelvic, and lower extremities injuries (*P* = 0.001 for all). MVCs were more likely to cause cervical spine injuries (*P* = 0.02) and their victims had higher rate of open reduction/internal fixation (*P* = 0.001). Rehabilitation was required mainly after FHO injuries (*P* = 0.007). Workers with burn injuries had significantly higher ICU (*P* = 0.04) and hospital length of stay (*P* = 0.004). The aggregate incidence of WRI in Qatar, from 2010 to 2012, was 49.27 per 100,000 workers and WRI mortality was 1.97 per 100,000 workers per year [12].

## 4. Discussion

This is the first report of occupational injury surveillance data to describe the epidemiology and outcome of serious WRI in Qatar. The analysis is based on the trauma registry of the only level I Trauma Center in Qatar; it is an accurate representation of all serious WRIs that occur in the country because all such cases are transferred to our center. The incidence and death rates of WRI in Qatar are much lower than the published reports from other similar countries in the region.

WRI represents a significant health and economic concern worldwide. However, the evaluation of the global burden of WRI and diseases remains challenging. In particular, developing nations lack reliable information on WRI which is mainly attributed to the limited resource allocation, negligence, and underreporting [3]. Most of the current literature on traumatic WRI is based mainly on reports from the developed countries.

According to the International Labor Organization (ILO), the global mortality (2008) from fatal occupational accidents was 321,000 [3]. In addition, around 317 million workers sustain nonfatal WRI annually, leading to an average loss of productivity of four days or more/year [3]. A high rate of WRI (1,980 per 100,000 workers per year) has been reported from Oman that has a socioeconomic status similar to Qatar [17]. The incidence of WRI among developing countries in Africa like Ghana is also high with an incidence of 1,150 per 100,000 workers per year in the urban areas and 4,490 per 100,000 workers per year in the rural areas [18].

Reports from USA showed a similar incidence of WRI in the high-risk Hispanic workers in Massachusetts (54.8 per 100,000 workers per year) and Washington State (45.5 per 100,000 workers per year) [19, 20]. Despite the reports in the popular press questioning worker rights and welfare in Qatar, this study reports WRI incidence and mortality rates that are much lower than those reported from UAE (136 per 100,000 workers per year) [11], UK (99 major injuries per 100,000 workers per year) [21], and Oman [17] and are equivalent to reports on high-risk Hispanic workers in USA [19, 20].

The Hamad Trauma Unit admitted about 5152 trauma patients over a 3-year period, of them WRI comprises 29% of the total admissions. Over a 10-year period, data from Washington State Trauma Registry in USA showed that WRI formed only 7.3% of all trauma admissions [20]. This study showed very similar results as the report by Barss et al. from the UAE, with 30% of all trauma admissions being due to WRI [11]. The relative burden of occupational trauma in Qatar is four times that in USA. This information should spur the formulation of policies to include initiatives for occupational safety and injury prevention as national priority issues for policy formulation, research, and programs.

Several epidemiological studies have observed a higher incidence of WRI among young males [1, 10, 17, 22, 23]. The Bureau of Labor Statistics in USA reported 5,488 workplace-related deaths in 2007, of which the majority were male workers (92%) [24]. Similarly, the majority of workers in this study were males with a mean age of 34 years. In this series, higher proportions of expatriate workers are from Nepal and India, who are involved in labor-intensive jobs such as building and road construction and industrial work. In a 2003 report on WRI in the neighboring UAE, 98% of victims were males, 74% were from South Asia, and 69% aged between 25 and 44 years [11]. This is similar to the situation in USA where a large percentage of immigrants work as laborers, of which 47% belong to ethnic minorities and only 15% were white with lower WRI rate. A report from Singapore showed that the majority of WRI occurred among nationals of China (53%), India (23%), and Bangladesh (14%). The number of occupational injuries is incidentally higher among minority group [25]. In our report, 66% of WRI patients were from South Asia.

In the present study, the most common MOI was FFH. Barss et al. reported the same experience in the UAE where 51% of their WRI patients were victims of falls from height, 29% of which were from considerable height [11]. Tuma et al. [10] showed that the incidence of fall injuries in Qatar was 86.7 per 100,000 workers causing a significant financial burden (average $15,735 per patient) on the health care system. Data from Singapore showed that the main MOI in the admitted WRI patients were FFH (66%) followed by FHO at work (21.9%) with an overall mortality rate of 4.7% [5]. The second most common MOI was FHO (18%), similar again to the report from UAE (15%) [11].

The proportion of WRI occurring in laborers and installation/repairmen contrasts with the findings of Mock et al. from Ghana [18]. They reported that most WRI happened in MVC's (12.7% drivers and 19.4% traders) in urban areas. An Australian report on fatal WRIs observed 374 deaths, a mortality rate of 1.93 per 100 000 workers, in 2010-2011. Of these, 59% were directly job-related, 29% were transport-related, and 12% were bystander fatalities [4]. The Annual Statistics Report (2010-2011) in UK found the most dangerous occupations to be construction, agriculture, and waste and recycling which represent the highest mortality rates (0.6 per 100 000 workers) [21]. This belies the marked difference in the most common occupational injury risk exposures between different settings, that is, the extent of construction in Qatar and UK.

Although, work-related burn injuries in our series were only 2% of all WRI, 17% of burn victims died and they constituted 9% of mortalities. Unfortunately, our database was unable to document the extent, grade, type, associated traumatic injuries, and cause of death among burn-related causalities. Analysis based on MOI also revealed that among housekeeping workers the percent of injuries due to burns was high and further analysis of the higher rate of deaths among these workers is needed to identify opportunities for improvements in primary, secondary, and/or tertiary prevention of burns-related deaths and injuries.

Data from the National Census of Fatal Occupational Injuries (2012) showed highest mortality among transportation incidents (41%), of which 16% involved pedestrians who were struck by vehicles [26]. The other common mechanisms were violence in the workplace (17%), contact with object or equipment (16%), fatal falls, slips, or trips (15%), and fire and explosion (3%). The present study found the highest number of WRI amongst construction laborers (42% of all WRIs) and transportation workers (18% of all WRI).

The overall case fatality was 3.7%, with the most fatalities from falls and the highest lethality being related to burn and pedestrians injuries at work. The variability in the mortality patterns by MOI and occupation can be attributable to differential exposures (i.e., housekeeping →kitchen →fires →burns versus road construction work →road exposure →pedestrian injury). The higher incidence of these potentially preventable injuries can be attributed to the lack of stringent safety regulations, consistent enforcement of these regulations, and the proper orientation and training of the expatriate workers. Our study confirmed this, as only 36% of WRI patients used PPE, while the remaining injured workers were not in compliance with safety regulations in the workplace. Also, the mortality was nonsignificantly higher among older workers as compared to other age groups. Our findings are supported by an earlier report from Centers for Disease Control and Prevention which showed that the mortality rates were higher among workers involved in transportation and material moving. Particularly, older workers (≥65 yrs) constituted a higher percentage (23%) of deaths [27]. Over 40% of WRI victims were dead on arrival which highlights the importance of primary injury prevention for occupational safety in Qatar.

In contrast to figures from the National Census of Fatal Occupational Injuries, victims of workplace violence did not make up a significant proportion of our patients with WRI.

Interestingly, our study observed an increased need for ventilatory support and prolonged ICU stay of WRI cases which reflects the severity of injury and financial burden on the healthcare system.

There are several limitations and areas for recommended improvement in our study such as underrepresentation of minor WRI as the trauma registry only encodes data from patients with severe injury or polytrauma, underreporting of protective measures used, deficient detailed medical history of patients, lack of data about medical insurance issues, work place ergonomics, work site environment, and employers' details (both private and public sectors). Also, language barriers represented a major limitation for medical staff communication with patients and subsequently the accuracy of documentation. Further, while our trauma registry data is prospectively encoded, retrospective exploration of this database shows that it still lacks certain WRI-specific data elements that are needed to fully inform policies and programs for occupational safety in Qatar. The creation of an integrated occupational injury registry with sources of data from the ambulance, emergency, hospital, and rehabilitation services in collaboration with the Ministry of Labor and insurance companies will mitigate the shortcomings of our present occupational surveillance system.

This study identified the most common and lethal mechanisms of WRI in Qatar.

FFH should be the focus of primary prevention efforts based on their high mortality and injury burden. Burn injuries in housekeeping workers, MVCs, and pedestrian injuries are other areas for prioritization.

Future research should focus on improving the quality of data on occupational injuries in Qatar. The creation of a dedicated multidisciplinary task force that will prospectively collect all data on risk factors and outcomes for WRI and link these with incident investigations from the Ministry of Labor is the first step needed. The investigation of the relationship between chronic conditions, the incidence and cost of WRI among high-risk occupations, workplace violence, and recurrent workplace injuries could be some of the priority issues this dedicated occupational injury registry will address. Therefore, occupational health and safety should be specifically tailored for the workers involved in most hazardous occupations and vulnerable groups. Strict governance from the Ministry of Labor is required to ensure compliance with safety measures as well as necessary precautions to avoid health risks from WRI in the workplace. There is an urgent need to improve the quality of data regarding WRIs in the rapidly developing high-income countries of the Middle East in order to ensure that progress is sustained but not at the expense of worker health and welfare. We also recommend the need for multiagency review of health provision for migrant workers, which should be based on thorough and independent evaluation for the major causes of mortality among migrant construction workers and identifying key measures to improve health and safety of workers in Qatar.

In conclusion, although the incidence of WRI in Qatar is quite substantial, its mortality rate is relatively low in comparison to other countries of similar socioeconomic status. The implementation and enforcement of strict safety regulations and use of PPE should be mandatory in workplaces, particularly in construction industry. WRIs cause a significant socioeconomic burden as evidenced by their extended length of hospital stay and prevalent need for rehabilitation services. The authors call for better evidence on the risk factors for WRI which must be used to formulate and implement focused injury prevention programs in the populations and industries identified to be at the highest risk for WRI.

## Figures and Tables

**Table 1:** Characteristics of injured workers and injuries sustained, Qatar Trauma Registry, 2010–2012.

	*n* = 1496 (%)
Males	1458 (97.5)
Age (mean ± SD; yrs)	34.3 ± 10.3
Age (range; yrs)	
<20	26 (1.8)
20–30	611 (41.3)
31–40	472 (32)
41–50	240 (16)
>50	130 (9)
Nationality	
Nepalese	437 (29)
Indian	307 (21)
Bangladeshi and Sri Lankan	238 (16)
Others	514 (34)
Mechanism of injury	
Fall from height	761 (51)
Motor vehicle crash	248 (16.6)
Struck by heavy object	268 (18)
Machinery	63 (4)
Burns	30 (2)
Pedestrians (during work)	34 (2.3)
Others	92 (6)
Type of work	
Laborers	632 (42.4)
Transport	271 (18)
Installation/repair	173 (12)
Carpenter	133 (9)
Housekeeping	39 (2.6)
Farming/fishing	24 (1.6)
Maintenance, ground cleaning	21 (1.4)
Forman	18 (1.2)
Others	185 (12.3)
No protective measures	956 (64)
Injury body region	
Head	307 (20.5)
Chest	385 (26)
Lower extremities	425 (28)
Upper extremities	347 (23)
Abdomen	276 (18)
Pelvis	207 (14)
Skin and soft tissue injuries	795 (53)
Lumbar Spine	308 (20.6 )
Thoracic Spine	161 (10.8 )
Cervical spine	92 (6.1 )
Procedures	
Open reduction and internal fixation	407 (27)
Exploration laparotomy	87 (6)
Craniotomy	44 (3)
Closed reduction	73 (5)
External fixation	36 (2.4)
Thoracotomy	9 (1)
ICU length of stay (median, range)	3 (1–64)
Hospital length of stay (median, range)	6 (1–192)
Ventilation days (median, range)	2 (1–21)
Rehabilitation needed	204 (13.6)
Prehospital death/brought in dead (BID)	22 (1.5)
Mortality	56 (3.7)

**Table 2:** Presentation of injury severity among injured workers.

	Mean ± standard deviation	Median; range
Injury severity score	11.7 ± 8.9	9 (1–75)
Glasgow coma score (scene)	14.03 ± 2.8	15 (3–15)
Head abbreviated injury score	3.4 ± 0.8	3 (1–5)
Chest abbreviated injury score	2.6 ± 0.7	3 (1–5)
Abdomen abbreviated injury score	2.5 ± 0.8	2 (1–5)
Pelvic abbreviated injury score	2.4 ± 0.7	2 (2–5)

**Table 3:** Case fatality rate and injury and mortality burden based on the mechanism of injury (Trauma Registry, Hamad Trauma Center, Doha, Qatar, 2010–2012).

	Fall from height	Motor vehicle crash	Fall of heavy objects	Machinery injury	Burn	Pedestrian injury
Injury burden (%)*	761/1496 (51)	248/1496 (17)	268/1496 (18)	63/1496 (4)	30/1496 (2)	34/1496 (2)
Mortality burden (%)**	21/56 (38)	15/56 (27)	8/56 (14)	2/56 (4)	5/56 (9)	4/56 (7)
Case fatality rate (per 100 cases)^•^	21/761 (3)	15/248 (6)	8/268 (3)	2/63 (3)	5/30 (17)	4/34 (12)

^∗,∗∗,•^For definitions, refer to the methodology section.

**Table 4:** Demography, presentation, and outcome by mechanism of injury.

	FFH *n* = 761 (%)	MVC *n* = 248 (%)	FHO *n* = 268 (%)	Machine *n* = 63 (%)	Burns *n* = 30 (%)	Pedestrians *n* = 34 (%)	*P* value
Males	735 (96.6)	244 (98.4)	267 (99.6)	62 (98.4)	27 (90)	33 (97.1)	0.02
Age (mean ± SD)	34.5 ± 10	34.3 ± 10.3	34.2 ± 10	34 ± 10.3	31 ± 8.6	34.7 ± 9.6	0.72
Nationality							0.001
Nepalese	235 (30.9)	51 (20.6)	85 (31.7)	21 (33.3)	8 (26.7)	14 (41.2)	
Indian	146 (19.2)	55 (22.2)	56 (20.9)	10 (15.9)	9 (30)	8 (23.5)	
Bangladeshi	68 (8.9)	19 (7.7)	23 (8.6)	7 (11.1)	1 (3.3)	1 (2.9)	
Sri Lankan	39 (5.1)	25 (10.1)	27 (10.1)	9 (14.3)	3 (10)	1 (2.9)	
Pakistani	26 (3.4)	27 (10.9)	12 (4.5)	3 (4.8)	1 (3.3)	0 (0)	
Philippine	30 (3.9)	5 (2)	9 (3.4)	5 (7.9)	2 (6.7)	4 (11.8)	
Egyptian	76 (10)	19 (7.7)	18 (6.7)	2 (3.2)	2 (6.7)	1 (2.9)	
Occupation							0.001
Laborers	378 (49.7)	12 (4.8)	157 (58.6)	40 (65.6)	2 (6.9)	18 (52.9)	
Transport	22 (2.9)	224 (90.3)	11 (4.1)	0 (0)	1 (3.4)	2 (5.9)	
Installation/repair	91 (12)	0 (0)	44 (16.4)	9 (14.8)	9 (31)	4 (11.8)	
Carpenter	101 (13.3)	0 (0)	22 (8.2)	4 (6.6)	0 (0)	2 (5.9)	
Housekeeping	33 (4.3)	0 (0)	0 (0)	0 (0)	4 (13.8)	0 (0)	
Farming/fishing	14 (1.8)	0 (0)	3 (1.1)	0 (0)	0 (0)	0 (0)	
Maintenance, ground cleaning	14 (1.8)	0 (0)	1 (0.4)	3 (4.9)	1 (3.4)	0 (0)	
No personal protective equipment (PPE)	562 (73.9)	102 (41.1)	182 (67.9)	40 (63.5)	16 (53.3)	11 (32.4)	0.001
Rehabilitation needed	107 (14.1)	37 (14.9)	47 (17.5)	2 (3.2)	1 (3.3)	5 (14.7)	0.007
Injured body region							
Head	183 (24)	42 (16.9)	51 (19)	6 (9.5)	0 (0)	7 (20.6)	0.002
Lower extremities	206 (27.1)	76 (30.6)	91 (34)	20 (31.7)	0 (0)	13 (38.2)	0.001
Chest	210 (27.6)	80 (32.3)	56 (20.9)	4 (6.3)	0 (0)	12 (35.3)	0.001
Upper extremities	201 (26.4)	67 (27)	39 (14.6)	17 (27)	1 (3.3)	9 (26.5)	0.001
Abdomen	139 (18.3)	62 (25)	45 (16.8)	4 (6.3)	0 (0)	10 (29.4)	0.001
Pelvis	114 (15)	36 (14.5)	44 (16.4)	0 (0)	0 (0)	9 (26.5)	0.001
Skin/soft tissue injuries	353 (46.4)	156 (62.9)	143 (53.4)	35 (55.6)	30 (100)	54 (76.6)	0.001
Cervical spine	54 (7.1)	23 (9.3)	12 (4.5)	1 (1.6)	0 (0)	1 (2.9)	0.02
Lumbar spine	223 (29.3)	24 (9.7)	52 (19.4)	0 (0)	0 (0)	6 (17.3)	0.001
Thoracic spine	107 (14.1)	22 (8.9)	27 (10.1)	1 (1.6)	0 (0)	3 (8.8)	0.001
ORIF	211 (27.7)	80 (32.3)	82 (30.6)	11 (17.5)	0 (0)	9 (26.5)	0.001
Exploration laparotomy	31 (4.1)	26 (10.5)	17 (6.3)	1 (1.6)	0 (0)	5 (14.7)	0.001
Injury severity score (mean ± SD)	12.02 ± 8	12.1 ± 9.8	11.8 ± 7.7	6.5 ± 4.8	—	13.9 ± 12.8	0.001
Intensive care unit days (median, range)	3 (1–52)	4 (1–36)	3 (1–59)	2 (1–5)	11 (1–64)	3.5 (1–22)	0.04
Hospital LOS (median, range)	6 (1–132)	7 (1–192)	7 (1–154)	4 (1–44)	9 (1–84)	6 (1–90)	0.004
Ventilation days (median, range)	2 (1–19)	2 (1–15)	2 (1–21)	2 (1-2)	2 (1–13)	1 (1–16)	0.87
Glasgow coma score (mean ± SD)	14.02 ± 3	13.7 ± 3.1	14.4 ± 2.3	13.8 ± 3.5	12.7 ± 4.6	14 ± 3.2	0.06
Brought in dead	9 (1.2)	5 (2)	2 (0.7)	2 (3.2)	2 (6.7)	2 (5.9)	0.02
Mortality	21 (2.8)	15 (6)	8 (3)	2 (3.2)	5 (16.7)	4 (11.8)	0.001

MVC: motor vehicle crash; FFH: fall from height, FHO: struck by heavy object; ORIF: open reduction and internal fixation; ISS: injury severity score; LOS: length of stay; Chi-Square test (categorical variables); one-way ANOVA (continuous variables).

## References

[1] Smith GS, Wellman HM, Sorock GS (2005). Injuries at work in the US adult population: contributions to the total injury burden. *The American Journal of Public Health*.

[2] Nagai R, Lefèvre AMC, Lefèvre F (2007). Knowledge and practices by adolescents in preventing occupational injuries: a qualitative study. *Revista de Saúde Pública*.

[3] (2011). Global trends and challenges on occupational safety and health. *ILO Introductory Report to the XIX World Congress on Safety and Health at Work*.

[4] Safe Work Australia Work-related Traumatic Injury Fatalities. http://www.ncis.org.au/wp-content/uploads/2013/04/External-Mortality-Data-Reports-WorkRelatedTraumaticInjuryFatalities2010-11.pdf.

[5] Ng ZX, Teo LT, Go KT, Yeo YT, Chiu MT (2010). Major workplace related accidents in Singapore: a major trauma centre’s experience. *Annals of the Academy of Medicine Singapore*.

[6] Schenker MB (2010). A global perspective of migration and occupational health. *The American Journal of Industrial Medicine*.

[7] Gubéran E, Usel M (1998). Permanent work incapacity, mortality and survival without work incapacity among occupations and social classes: a cohort study of ageing men in Geneva. *International Journal of Epidemiology*.

[8] Turner JA (2000). Predictors of chronic disability in injured workers. *The American Journal of Industrial Medicine*.

[9] Jeong BY (1998). Occupational deaths and injuries in the construction industry. *Applied Ergonomics*.

[10] Tuma MA, Acerra JR, El-Menyar A (2013). Epidemiology of workplace-related fall from height and cost of trauma care in Qatar. *International Journal of Critical Illness and Injury Science*.

[11] Barss P, Addley K, Grivna M, Stanculescu C, Abu-Zidan F (2009). Occupational injury in the United Arab Emirates: epidemiology and prevention. *Occupational Medicine*.

[12] Atique S, Zarour A, Siddiqui T (2012). Trauma caused by falling objects at construction sites. *Journal of Trauma and Acute Care Surgery*.

[13] Qatar Statistics Authority Labor force sample survey: annual report: 2011 Doha, Qata. http://www.qsa.gov.qa/eng/publication/pdf-file/Social/Labor%20Force%20Report%202011.pdf.

[14] Amnesty International The Dark Side of Migration: Spotlight On Qatar's Construction Sector Ahead of The World Cup. https://www.amnesty.org.uk/sites/default/files/the_dark_side_of_migration_-_spotlight_on_qatars_construction_sector_ahead_of_the_world_cup.pdf.

[15] Nivedita Ram Health, safety and environment procedure: Incident Notification, Analysis, Reporting and Follow-Up. *PDO HSE Documents*.

[16] Baker SP, O’Neill B, Haddon W, Long WB (1974). The injury severity score: a method for describing patients with multiple injuries and evaluating emergency care. *Journal of Trauma*.

[17] Al-Rubaee FR, Al-Maniri A (2011). Work related injuries in an oil field in Oman. *Oman Medical Journal*.

[18] Mock C, Adjei S, Acheampong F, Deroo L, Simpson K (2005). Occupational injuries in Ghana. *International Journal of Occupational and Environmental Health*.

[19] Hunt PR, Won JU, Dembe A, Davis L (2005). Work-related hospitalizations in Massachusetts: racial/ethnic differences. *Monthly Labor Review*.

[20] Sears JM, Bowman SM, Adams D, Silverstein BA (2011). Occupational injury surveillance using the Washington state trauma registry. *Journal of Occupational and Environmental Medicine*.

[21] Health and Safety Executives Annual Statistics report 2010/11. http://www.hse.gov.uk/statistics/overall/hssh1011.pdf.

[22] Noe R, Rocha J, Clavel-Arcas C, Aleman C, Gonzales ME, Mock C (2004). Occupational injuries identified by an emergency department based injury surveillance system in Nicaragua. *Injury Prevention*.

[23] Righi E, Gatti G, Marcheselli R, Fantuzzi G, Aggazzotti G (2003). Epidemiology of work related injuries in young people: results of a survey carried out in Modena (Italy) between January and June 2000. *Annali di Igiene*.

[24] US Department of Labor, Bureau of Labor Statistics National census of fatal occupational injuries in 2007. http://www.cdc.gov/niosh/programs/ptdesign/sector.html.

[25] Aguirre-Molina M, Molina CW (1990). Ethnic/racial populations and worksite health promotion. *Occupational Medicine*.

[26] Bureau of Labour Statistics National Census of Fatal Occupational Injuries in 2012. http://www.bls.gov/news.release/pdf/cfoi.pdf.

[27] Older workers who drive top traffic death list: CDC. http://www.nlm.nih.gov/medlineplus/news/fullstory_139995.html.

